# Exploring Physical and Psychosocial Well-Being and Self-Awareness as a New Frontier in Active Aging

**DOI:** 10.2174/1745017901814010294

**Published:** 2018-11-29

**Authors:** Federica Sancassiani, Donatella Rita Petretto, Ferdinando Romano, Antonio Preti

**Affiliations:** 1 Department of Medical Sciences and Public Health, University of Cagliari, Cagliari, Italy; 2 Department of Psychology, University of Cagliari, Cagliari, Italy; 3 Department of Public Health and Infectious Diseases, University of Rome “La Sapienza”, Rome, Italy

The knowledge about the effects of exercise, physical and sport activities on general well-being has been advanced thanks to pioneering studies in several medical conditions and in rehabilitation from the 1980s onwards [1]. However, a noteworthy contribution to improving standard tools hallowing to measure of how much exercise, physical and sport activities could affect the quality of life (QoL) of the elderly and adults came mainly from the studies on their effects on depression and mental health [2-4].

First, the new perspectives have made it possible to establish the level of QoL, which cannot be considered as a simple variable depending on the state of health: actually, there are consolidated evidence of a complex relationship between health, QoL, exercise, physical and sport activities [5, 6]. Second, this complex vision of physical and psychosocial health has benefited from the concepts of well-being and QoL applied, first of all, like “extended” outcome measures to the studies on the use of exercise, physical and sport activities in depression [7, 8].

However, the challenge of active aging today can be placed in a new syncretic dimension in which aging is not seen (only) as the product of the progressive impairment to the body due to age, and not (only) due to social and architectural, physical and other types of barriers that prevent the person who advances with age to enjoy the maximum possible functioning. Active aging is a process of awareness of one's own limitations and, above all, of one's own resources that allow to cope with the barriers in the most functional way [9].

Within this perspective, subjectivity is central, that is why the measure of body awareness as explored by “The Physical Body Experiences Questionnaire Simplified for Active Aging (PBE-QAG)” [10] is a key dimension.

This questionnaire can undoubtedly be improved. For example, a comparison could be introduced in a more extended version of how the person feels the body today and how she felt it when she was younger, *e.g.*, 40 years earlier.

Nevertheless, it should be emphasized that since it is the first instrument to address the measurement of such an important dimension, we think that this work represents a milestone.
